# Allosteric regulation by membranes and hydrophobic subsites in phospholipase A_2_ enzymes determine their substrate specificity

**DOI:** 10.1016/j.jbc.2022.101873

**Published:** 2022-03-28

**Authors:** Edward A. Dennis

**Affiliations:** Department of Chemistry and Biochemistry and Department of Pharmacology, School of Medicine, University of California at San Diego, La Jolla, California, USA

**Keywords:** allosterism, specificity, phospholipase, enzyme mechanism, membrane, micelle, lipid metabolism, inflammation, AA, arachidonic acid, cPLA_2_, cytosolic phospholipase A_2_, DXMS, deuterium exchange mass spectrometry, iPLA_2_, calcium-independent phospholipase A_2_, Lp-PLA_2_, lipoprotein-associated PLA_2_, MD, molecular dynamics, PAF, platelet-activating factor, PAPC, *sn*-1 palmitoyl, *sn*-2 arachidonyl phosphatidylcholine, PAPE, 1-palmitoyl, 2-arachidonyl phosphatidylethanolamine, PLA_2_, phospholipase A_2_, PLPC, *sn*-1 palmitoyl, *sn*-2 linoleoyl phosphatidylcholine, PNPLA, patatin-like PLA_2_, POPC, *sn*-1 palmitoyl, sn-2 oleoyl phosphatidylcholine, sPLA_2_, secreted phospholipase A_2_

## Abstract

Lipids play critical roles in several major chronic diseases of our times, including those that involve inflammatory sequelae such as metabolic syndrome including obesity, insulin sensitivity, and cardiovascular diseases. However, defining the substrate specificity of enzymes of lipid metabolism is a challenging task. For example, phospholipase A_2_ (PLA_2_) enzymes constitute a superfamily of degradative, biosynthetic, and signaling enzymes that all act stereospecifically to hydrolyze and release the fatty acids of membrane phospholipids. This review focuses on how membranes interact allosterically with enzymes to regulate cell signaling and metabolic pathways leading to inflammation and other diseases. Our group has developed “substrate lipidomics” to quantify the substrate phospholipid specificity of each PLA_2_ and coupled this with molecular dynamics simulations to reveal that enzyme specificity is linked to specific hydrophobic binding subsites for membrane phospholipid substrates. We have also defined unexpected headgroup and acyl chain specificity for each of the major human PLA_2_ enzymes, which explains the observed specificity at a structural level. Finally, we discovered that a unique hydrophobic binding site—and not each enzyme’s catalytic residues or polar headgroup binding site—predominantly determines enzyme specificity. We also discuss how PLA_2_s release specific fatty acids after allosteric enzyme association with membranes and extraction of the phospholipid substrate, which can be blocked by stereospecific inhibitors. After decades of work, we can now correlate PLA_2_ specificity and inhibition potency with molecular structure and physiological function.

The ASBMB 2021 Bert and Natalie Vallee Award in Biomedical Sciences honors our work on how phospholipase A_2_ (PLA_2_) acts on substrate in membranes and micelles. Lipids play critical roles in the metabolic syndrome including obesity, insulin sensitivity and type 2 diabetes, numerous cardiovascular diseases, and fatty liver disease including nonalcoholic steatohepatitis; these constitute the major chronic diseases of our times, and all of these involve sequelae of inflammation. Over 47,000 distinct molecular species of lipids have been identified by the LIPID MAPS Consortium (www.lipidmaps.org), so defining substrate specificity of enzymes of lipid metabolism is a challenging task.

Over the years, our laboratory (1) discovered and demonstrated that membranes interact allosterically with enzymes to regulate cell signaling and metabolic pathways leading to inflammation (2). We have recently employed “substrate lipidomics” coupled with molecular dynamics (MD) to reveal enzyme specificity linked to highly specific hydrophobic binding sites or “subsites” for the *sn-2* fatty acyl chains in membrane phospholipid substrates (3). We discovered unexpected headgroup and acyl chain specificity for each of the major human phospholipase A_2_ enzymes that explains the observed specificity at a new atomic level. A unique hydrophobic binding site—and not each enzyme’s catalytic residues or polar headgroup binding site—dominates each enzyme’s specificity. Each PLA_2_ shows unique specificity for its required fatty acid ranging from proinflammatory omega-6 arachidonic acid (AA) or anti-inflammatory fish oil omega-3 eicosapentenoic acid and docosahexenoic acid; others favor membrane remodeling linoleic acid, antibacterial saturated fatty acids, or oxidized fatty acids in low-density lipoproteins. Each PLA_2_ releases a specific fatty acid after the enzyme associates with membranes causing an allosteric effect and extracts a single phospholipid substrate into its catalytic site. Stereospecific inhibitors (4) have been developed for the specific sites of each enzyme and studied kinetically. After decades of advances in lipid research, we can now correlate PLA_2_ specificity and inhibition potency with molecular structure and physiological function using a novel lipidomics platform that provides a paradigm for protein–membrane lipid interactions in general.

## PLA_2_ superfamily

**Figure 1:**
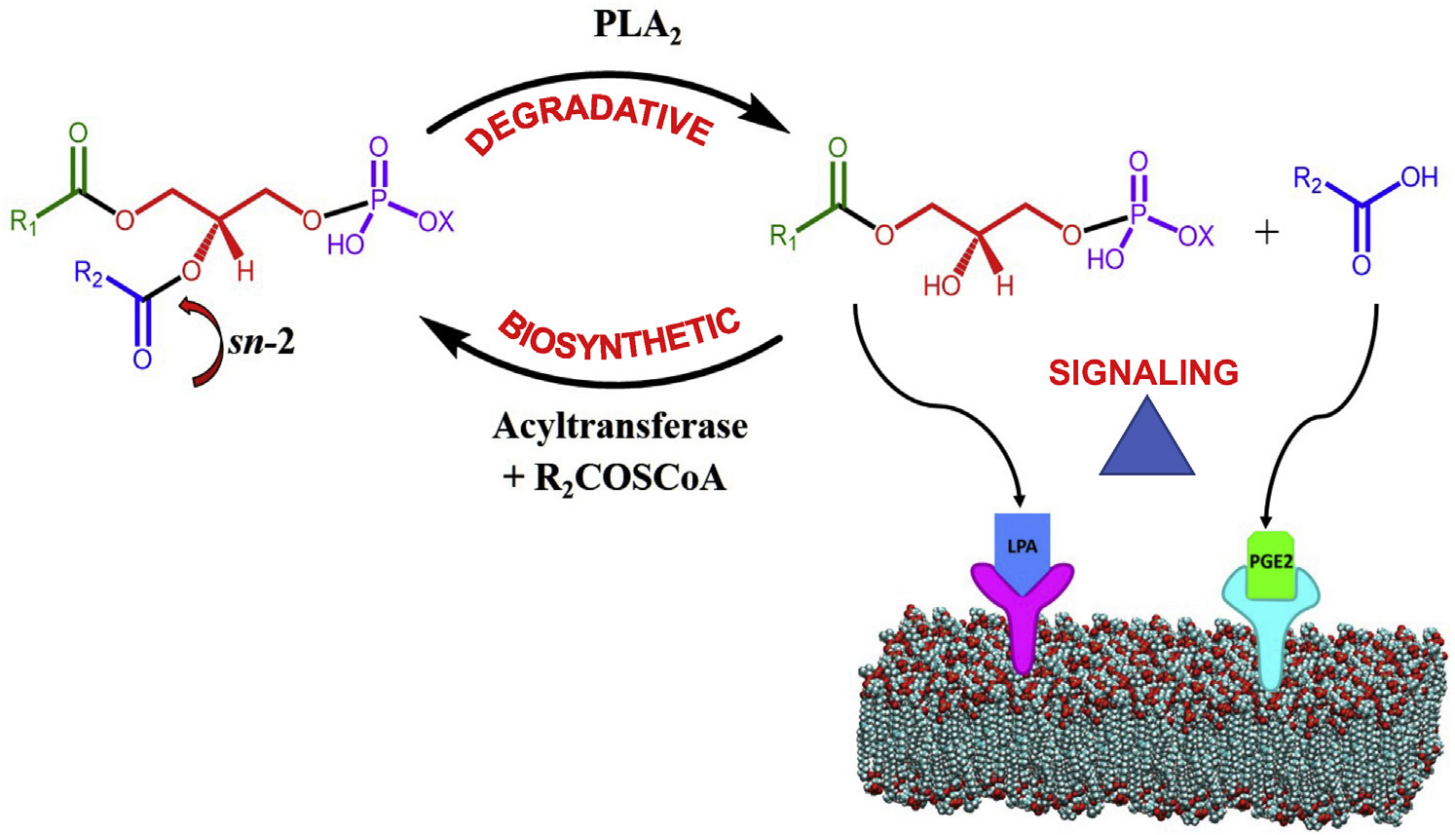
**Phospholipases A**_**2**_**are degradative, biosynthetic, and signaling enzymes.** They all specifically hydrolyze the fatty acid on the *sn*-2 position, but the primary purpose of some of them is degradative, producing a lysophospholipid and free fatty acid, while others are biosynthetic and are often coupled with one of the many acyl transferases to add a PUFA to the *sn*-2 position. Others serve as signaling enzymes coupled with a variety of downstream enzymes as pictured here with a lysophospholipase D to produce lysophosphatidic acid (LPA) or with a cyclooxygenase and a prostaglandin synthase to produce prostaglandin E2 (PGE_2_). PUFA, polyunsaturated fatty acid. Adapted from the study by Dennis *et al.* (5).

PLA_2_s constitute a superfamily of enzymes: some are degradative, some are biosynthetic, and others are signaling enzymes (Fig. 1) (5). All of the PLA_2_s act stereospecifically to hydrolyze the fatty acid at the middle or *sn-2* position of membrane phospholipids. When they produce as products lysophospholipids and free fatty acids, they are degradative enzymes. When the enzyme is coupled to an acyltransferase that specifically puts a polyunsaturated fatty acid back in that *sn-2* position to make a new phospholipid that has been remodeled, then they “in effect” become biosynthetic enzymes. When the products are converted by other enzymes to ligands that activate G protein–coupled receptors such as lyso-phosphatidic acid or PGE_2_, then they are considered signaling enzymes.

The *Group Numbering System* was originally developed to differentiate the PLA_2_s that had been described at the time (6), and it was subsequently expanded (7) as more unique PLA_2_s were discovered, so it roughly reflects the order in which the different PLA_2_s were discovered. Today, there are over 16 *Groups* and many *Subgroups* that are comprised of distinct PLA_2_s (5). For simplicity, the PLA_2_s are sometimes referred to by general names as belonging to one of six *Types* using their more generic names (also commonly listed in the order that they were characterized): secreted phospholipase A_2_ (sPLA_2_); cytosolic phospholipase A_2_ (cPLA_2_); calcium-independent phospholipase A_2_ (iPLA_2_); platelet-activating factor (PAF) acetyl hydrolase, also known as lipoprotein-associated PLA_2_ (Lp-PLA_2_), lysosomal PLA_2_, and adipose PLA_2_, but if one wants to be specific about the particular PLA_2_, the designation by *Type* would then be preceeded by the *Group Number* (5, 6, 7). Numerous laboratories around the world, but particularly in Japan, France, the Netherlands, and the United States, contributed to this early development, and complete references to the initial discovery of each of these *Types* and authoritative reviews by others on the major *Groups* and *Types* are listed elsewhere (5) (complete with 532 references).

It should be noted that alternate naming systems have been used by many laboratories including first listing the *Type* followed by a Greek letter to indicate the specific *Group/Subgroup* (*i.e.*, most commonly cPLA_2_α instead of Group IVA (GIVA) and iPLA_2_β instead of Group VIA). Another naming system was developed independently for iPLA_2_ which describes enzymes based on sequence similarities rather than on catalytic substrate similarities based on a classic well-studied potato (patatin) triacylglycerol lipase resulting in “patatin-like PLA_2_” (PNPLA) for which Group VIA iPLA_2_ is known as PNPLA9. Other PNPLA enzymes actually exhibit different activities than PLA_2_, for example, PNPLA3 is actually a triacylglycerol lipase rather than a PLA_2_, and there is enormous interest in this enzyme currently because of its association with nonalcoholic fatty liver disease and its advanced form nonalcoholic steatohepatitis (8).

**Figure 2:**
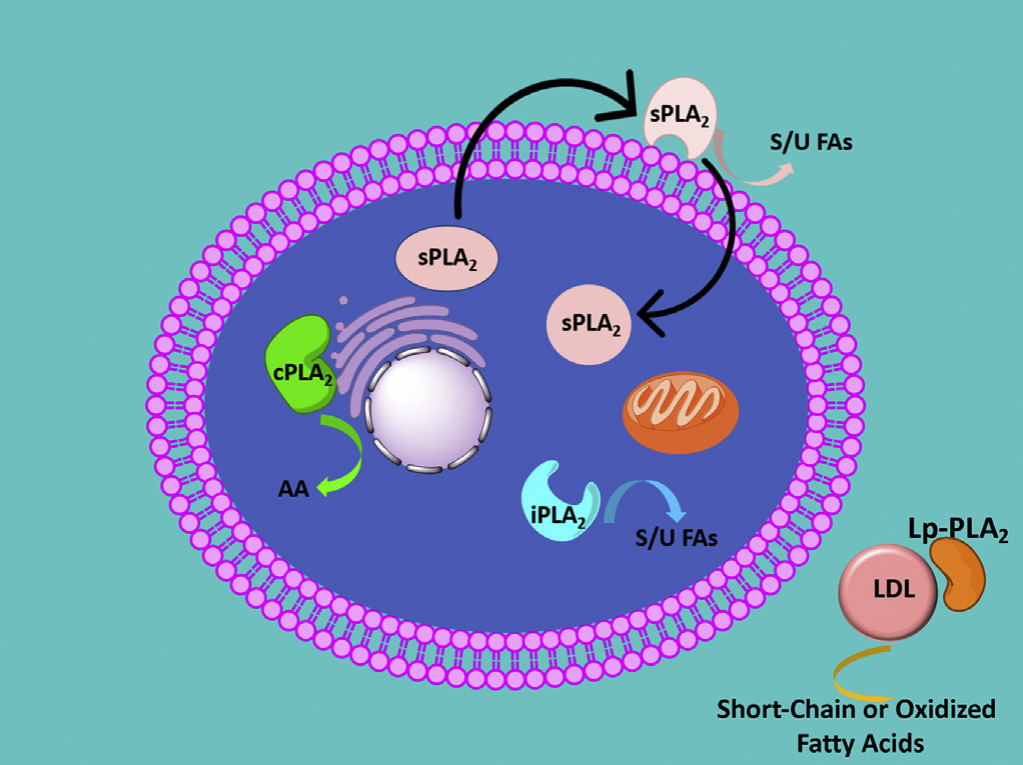
**Cellular localization and specificity of major phospholipase A**_**2**_**types.***Cartoon* showing the four major types of PLA_2_ discussed in this review. Group IVA cPLA_2_ is a cytosolic enzyme that upon cellular activation localizes to the Golgi or nuclear envelop in some cells and has a high specificity for releasing arachidonic acid from phospholipids. Group VIA iPLA_2_ is found in the cytosol and sometimes associated with mitochondria and releases saturated and unsaturated fatty acids. Group V sPLA_2_ is secreted from cells and acts on the outside of cells, but it has been suggested that it can also be reincorporated into cells through endocytic processes whereby it may also be active in the lumen of some intracellular organelles. It also releases saturated and unsaturated fatty acids. Group VIIA Lp-PLA_2_ is found associated with lipoproteins in the blood stream where it hydrolyzes the phospholipids which comprise the outer monolayer of the lipoprotein particle, but it will not hydrolyze normal long-chain saturated or unsaturated fatty acids but rather has a specificity for phospholipids containing an oxidized or a short-chain fatty acid as in platelet-activating factor (PAF) on its sn-2 position. cPLA_2_, cytosolic phospholipase A_2_; iPLA_2_, calcium-independent phospholipase A_2_; LDL, low-density lipoprotein; Lp-PLA2, lipoprotein-associated PLA_2_; PLA_2_, phospholipid A_2_; sPLA2, secreted phospholipase A_2_. Adapted from the studies by Shirai *et al.* (9), Cao *et al.* (10), and Mouchlis *et al.* (11).

This review of recent work in our laboratory will focus on the four major human phospholipase A_2_ *Types* studied, but each of these four are the major studied human form in their *Group* and have been among the most well-studied and well-characterized examples. They are all highly purified recombinant human enzymes that we study *in vitro* in our laboratory. The four enzymes and their subcellular localization are depicted in Figure 2 (9, 10, 11). The first is the GIVA cPLA_2_ which associates with the Golgi and specifically releases AA, a proinflammatory fatty acid that leads to inflammation and has a major role in signaling. Another PLA_2_ that is localized in the cytosol, but is sometimes associated with mitochondria, is the Group VIA iPLA_2_; it releases unsaturated fatty acids and is heavily involved in membrane remodeling and implicated in mitochondrial functioning.

As has been noted, there are multiple forms (designated as *Groups* and *Subgroups*) of each of the four *Types* of PLA_2_ discussed here, but the secreted PLA_2_ *Type* includes the largest number of well-studied distinct *Groups* and *Subgroups*. The first PLA_2_s to be characterized were secreted enzymes from various snake venoms and porcine pancreas, and the human Group IIA, which was originally cloned from human synovial fluid, is perhaps the most well studied (5, 12, 13). The secreted enzymes are formed in the Golgi and are secreted to the outside of cells where they act to release both saturated and unsaturated fatty acids. It has also been suggested that sometimes they undergo endocytosis and may act intracellularly as well. In this review, we have limited ourselves to discussing the Group V sPLA_2_ because among the sPLA_2_s, it has been the focus in our laboratory. Interestingly, it is secreted by macrophage cells (which also express the intracellular GIVA cPLA_2_ and Group VIA iPLA_2_), and macrophages additionally secrete the Lp-PLA_2_. The Group VIIA Lp-PLA_2_ is the fourth enzyme *Type* we have studied extensively. It is known as the secreted Lp-PLA_2_, and it associates with low-density lipoproteins and high-density lipoproteins in the blood stream and specifically releases only oxidized or short-chain fatty acids. This enzyme was independently discovered and named as PAF acetyl hydrolase because of its potent ability to hydrolyze PAF releasing acetate from the *sn*-2 position (5, 14).

## Surface dilution kinetics

**Figure 3:**
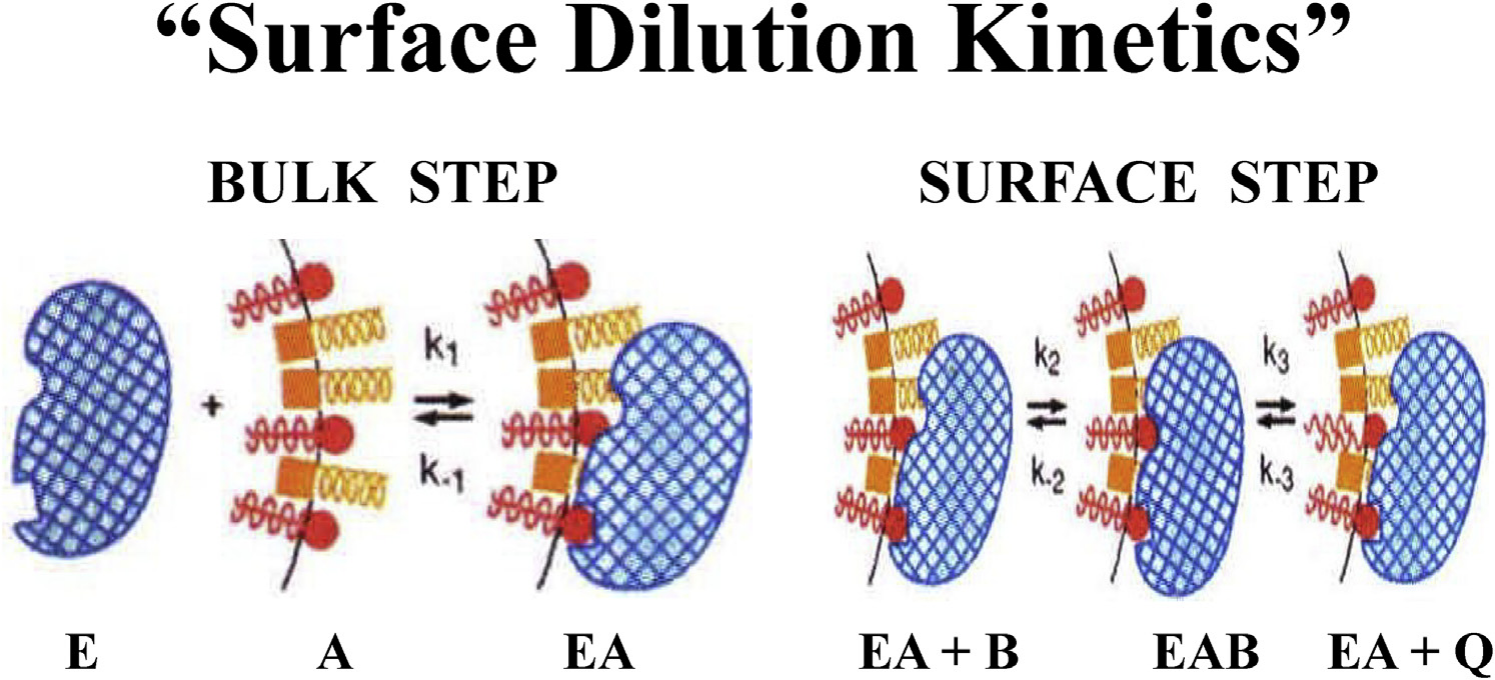
**Surface dilution kinetics.** Schematic view of water-soluble enzymes (E) such as phospholipase A_2_ (*blue*) associating with the lipid–water interface (A) to form the enzyme–interface (EA) complex which consists of phospholipid substrates (*red*) in bilayer membranes or mixed micelles with detergents/surfactants such as the nonionic surfactant Triton X-100 (*yellow*) or naturally occurring surfactants such as the bile acids at the lipid–water interface. The enzyme first associates with the mixed micelle surface (*Bulk Step*) forming the EA complex. Then in a subsequent step (*Surface Step*), the enzyme associated with the micelle (EA) extracts and binds a single phospholipid substrate molecule from the two-dimensional interface (B) in its catalytic site forming the EAB complex. The enzyme then carries out hydrolysis while still associated with the interface (EA complex), producing as products (Q) a lysophospholipid and a free fatty acid, which may be released to solution or may be retained in the micelle surface. Reprinted from the study by Carman *et al.* (17) and adapted from the studies by Dennis (15) and Deems *et al.* (16).

We recognized very early on in our initial studies in the early 1970s (15, 16) that if we are going to study PLA_2_s, and at that time we were focused on the secreted PLA_2_ purified from cobra venom (*Naja naja naja*), that since it was water soluble, it is going to interact with a phospholipid substrate which resides only in membranes or micelles and not freely in solution. Thus, the enzyme must first associate with the lipid aggregate and the first step for a phospholipase to act requires that the water-soluble enzyme associate with the membrane or the micelle. In Figure 3, the lipid–water interface is pictured as a mixed micelle with phospholipids in red and nonionic surfactant Triton-X100 molecules in yellow, though the same principle holds for bilayer membranes or phospholipid vesicles as well as the monolayer surface of lipoprotein particles and lipid droplets.

The four PLA_2_ enzymes we will discuss herein are all water soluble. The first step depends on the concentration of enzyme and membranes or micelles. Note that the phospholipids in red laterally diffuse around the surface of the micelle or membrane very rapidly until a single phospholipid is “sucked” into the catalytic site. Catalysis occurs, and the products diffuse back around the surface of the micelle or membrane. The second step occurs when the enzyme is associated with the surface, and this step depends kinetically on the surface concentration of the specific phospholipid substrate in the surface of the micelle. This realization led our group to propose “surface dilution kinetics” (15, 16) to analyze water-soluble enzymes acting on substrates localized to the lipid–water interface. This conceptual approach can be applied to other proteins that associate with membranes and especially to other enzymes of lipid metabolism (17) and seems equally applicable to substrates located on the outer surface of bilayer membranes, micelles, lipoproteins, lipid droplets, etc.

## Deuterium exchange mass spectrometry applied to protein–membrane and protein–substrate interactions

About a decade ago, our laboratory proposed using the technique of deuterium exchange mass spectrometry (DXMS) to look specifically for the first time at the interaction of proteins with individual phospholipid substrates and their interactions with the membrane (18).

Quite simply, one takes the PLA_2_ in aqueous solution, then mixes it with phospholipid vesicles, allows them to associate, dilutes in D_2_O, and then measures the rate of deuterium exchange with the amide protons in the amino acids in separate peptides in the polypeptide backbone of the enzyme. In essence, where the enzyme interacts with the membrane or where a phospholipid is pulled up into the enzyme active site, the rate of deuterium exchange is changed, both by proximity and by conformational changes that can be quite complicated. Peptides show increases and others show decreases in deuterium exchange depending on whether that portion of the enzyme is more exposed or less exposed to solvent. However, decreases are especially noteworthy when they result from decreased accessibility due to hydrophobic interactions (10).

**Figure 4:**
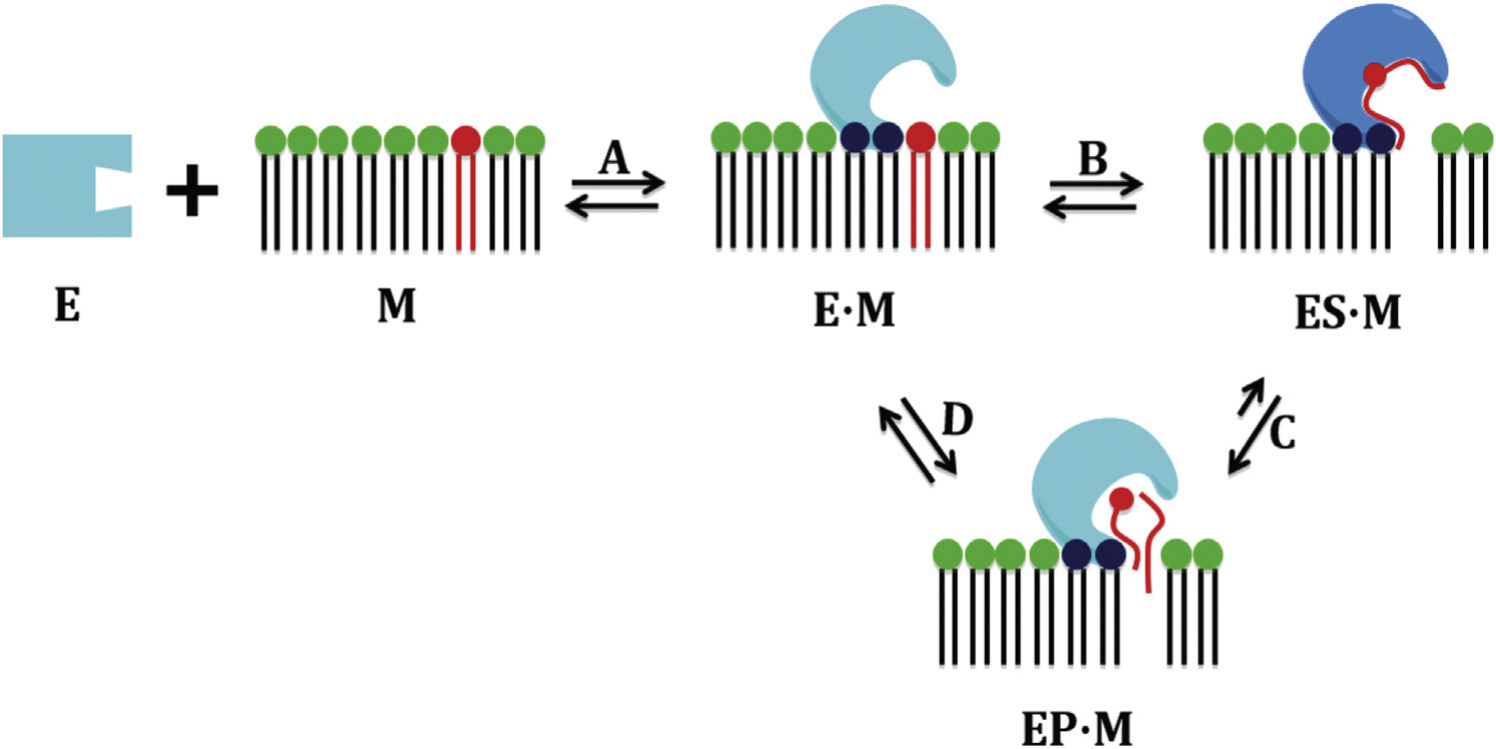
**Membrane allostery and unique hydrophobic sites promote enzyme substrate specificity.***Step A*, the water-soluble phospholipase A_2_ enzyme (E) associates with one side of a phospholipid bilayer membrane (M) to form a complex (E-M) whereby the phospholipid membrane acts at an allosteric site on the surface of the enzyme inducing a conformational change in the enzyme. *Step B*, the enzyme can then extract a single phospholipid substrate (S) from the bilayer membrane to which it is associated (ES-M) binding the polar portion (*red circle*) in its polar subsite and the *sn*-1 and *sn*-2 chains (*red*) in their specific subsites. *Step C*, catalysis occurs forming the lysophospholipid and free fatty acid products (P) while still associated to the membrane (EP-M complex). *Step D*, the products dissociate from the active site into the membrane (and then into the aqueous phase depending on their composition), and the enzyme repeats the cycle (E-M complex) with another substrate. Adapted from the study by Mouchlis *et al.* (3).

These studies led us to propose that membranes cause allosteric changes in the enzyme when they associate and that this facilitates catalysis as described elsewhere (19). We created the scheme (3) shown in Figure 4 that applies to all four of the enzymes under discussion. Quite simply, the water-soluble enzyme, when mixed with a phospholipid vesicle, causes a conformational change in the enzyme indicated by a square to a circle. Note that this very large membrane is antithetical to the original proposal of allosterism by Monod, Wyman, and Changeux (20), which was pictured as a small allosteric site, for example, with O_2_ binding to other subunits in tetrameric hemoglobin or perhaps a Ca^2+^ binding to another site causing a conformation change in a large enzyme. In the case of membranes, it is a very large membrane that causes a conformational change in a relatively much smaller protein as illustrated in Figure 4.

Once associated, the phospholipid molecules move very rapidly by lateral diffusion around the surface of the membrane until a single one is “sucked” into the catalytic site as shown, catalysis occurs, the products diffuse back in the membrane, and the cycle keeps repeating itself. We have suggested that this model is a general model applicable to all four of the PLA_2_ enzymes discussed herein and perhaps other membrane-associated enzymes as well.

## Cytosolic PLA_2_

**Figure 5:**
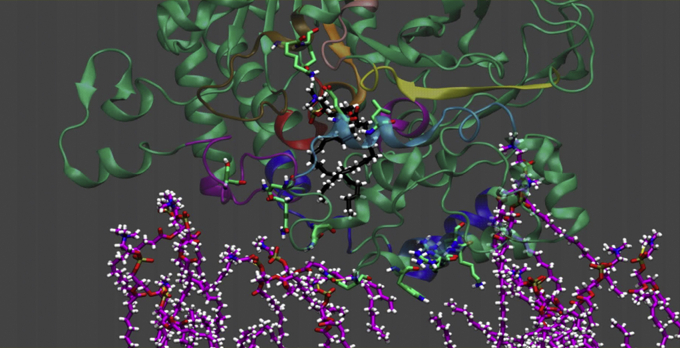
**Molecular dynamics simulation of GIVA cPLA**_**2**_**with PAPC substrate and POPC membrane.** Screen shot at the beginning of the simulation with the *sn*-1 palmitoyl, *sn*-2 arachidonyl phosphatidylcholine (PAPC) substrate docked in the active site and the enzyme docked on the *sn*-1 palmitoyl, sn-2 oleoyl phosphatidylcholine (POPC) as determined from deuterium exchange mass spectrometry (DXMS) experiments. See Movie S1 for complete simulation over 300 ns. cPLA_2_, cytosolic phospholipase A_2_. Adapted from the study by Mouchlis *et al.* (19).

In Figure 5, the catalytic domain of human GIVA cPLA_2_ was placed in a cube of water and a substrate *sn*-1 palmitoyl, *sn*-2 arachidonyl phosphatidylcholine (PAPC) molecule in black was docked in its active site and the cPLA_2_ was docked to a membrane patch as suggested by the DXMS results. The membrane patch consisted of a full *sn*-1 palmitoyl, sn-2 oleoyl phosphatidylcholine (POPC) bilayer membrane in purple (although only a slice of the membrane is shown, so the enzyme and substrate can be seen clearly). MD simulations were carried out for 300 ns. See Movie S1.

The first thing one notices is how rapidly the purple phospholipids in the membrane move with lateral diffusion, but basically staying oriented. In contrast, the phospholipid in the catalytic site is much more restricted in motion as is the whole protein as it struggles to find the minimal energy conformation of both the phospholipid ligand and the enzyme. The AA is seen in its characteristic curved form in its final conformation with its four *cis* double bonds interacting primarily with aromatic amino acid side chains with π-π stacking.

**Figure 6:**
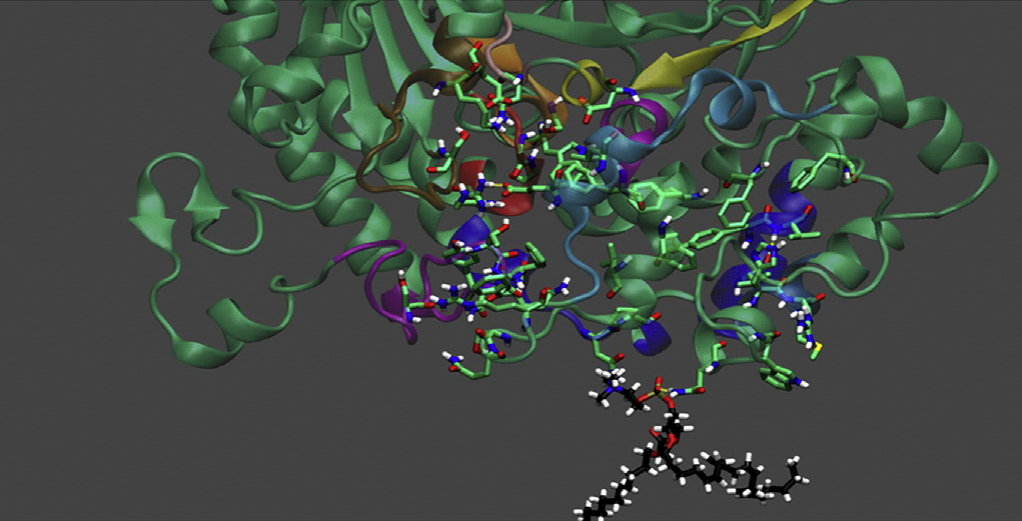
**Extraction of PAPC substrate into the active site of cPLA**_**2**_**.** Screen shot at the beginning of the simulation where the PAPC substrate is pulled out of the active site into the bilayer membrane along a trajectory, and then a slight force is applied in the opposite direction to cause re-entry along the same trajectory. See Movie S2 for complete simulation over 300 ns. cPLA_2_, cytosolic phospholipase A_2_; PAPC, *sn*-1 palmitoyl, *sn*-2 arachidonyl phosphatidylcholine. Adapted from the study by Mouchlis *et al.* (19).

In a separate experiment, the PAPC substrate in its final energy minimized docked conformation was pulled out of the active site along a straight trajectory back into the membrane, and then a slight force was applied in the opposite direction to allow this phospholipid to re-enter the active site. This allows one to visualize how the enzyme, docked to a membrane, might pull a single phospholipid out of the membrane and into the catalytic site. See Figure 6 and Movie S2. Note that the red helix in the protein houses the Ser–Asp dyad that is the catalytic machinery and the Ser is right adjacent to the *sn-2* carbonyl position oriented to initiate the catalytic event.

## Calcium-independent PLA_2_

**Figure 7:**
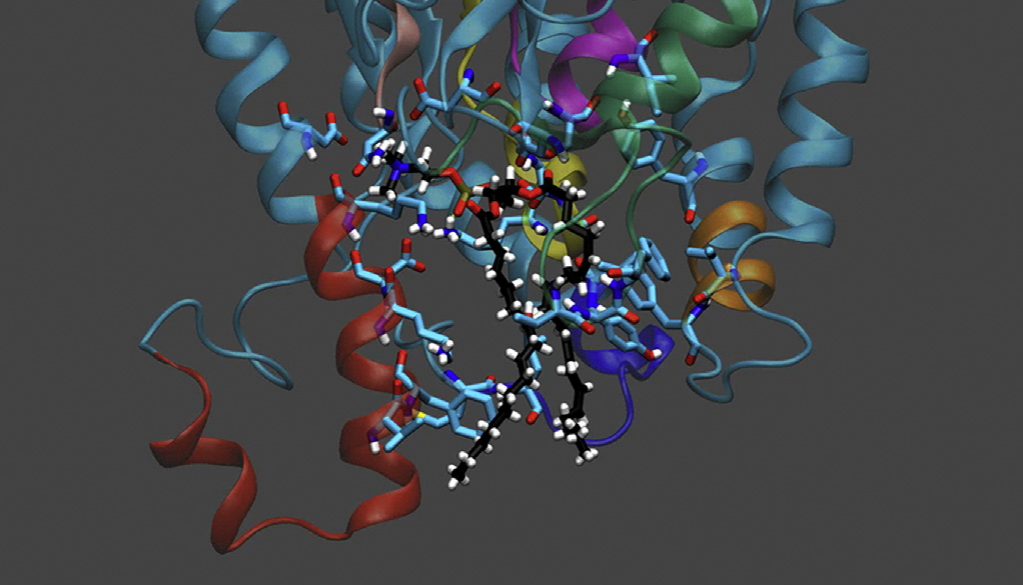
**Molecular dynamics simulation of GVIA iPLA**_**2**_**with PAPC substrate and POPC membrane.** Screen shot at the beginning of the simulation with the *sn*-1 palmitoyl, *sn*-2 arachidonyl phosphatidylcholine (PAPC) substrate docked in the active site and the enzyme docked on the *sn*-1 palmitoyl, sn-2 oleoyl phosphatidylcholine (POPC) (POPC not shown so can visualize the more open conformation of the iPLA_2_ active site) as determined from deuterium exchange mass spectrometry (DXMS) experiments. See Movie S3 for complete simulation over 300 ns. iPLA_2_, calcium-independent phospholipase A_2_. Adapted from the study by Mouchlis *et al.* (19).

The human Group VIA iPLA_2_ is a very different enzyme, though it has the same Ser–Asp dyad as its catalytic machinery. In Figure 7, we have docked the catalytic domain of iPLA_2_ with the same PAPC substrate as employed with cPLA_2_ in its catalytic site as well as the same POPC membrane patch and then carried out MD simulations for 300 ns. See Movie S3. The active site of iPLA_2_ is a little bit more of an open site than cPLA_2_, and in the middle of the simulation, one observes a conformational change whereby the enzyme opens up to accommodate the *sn-2* fatty acid chain, in this case AA, in a distinct subsite that binds very specifically to the *sn-2* fatty acid. The *sn-1* fatty acid, palmitate in this case, is less restricted, and it appears to extend some into the membrane patch. Note that the red helix on the enzyme is buried in the membrane, so one might visualize the membrane as allowing the tail of the *sn-1* chain to protrude into the membrane, whereas the *sn-2* fatty acid, which is the leaving or cleaved fatty acid, is bound entirely in its *sn-2* subsite.

## Lipidomics and MD simulations

**Figure 8:**
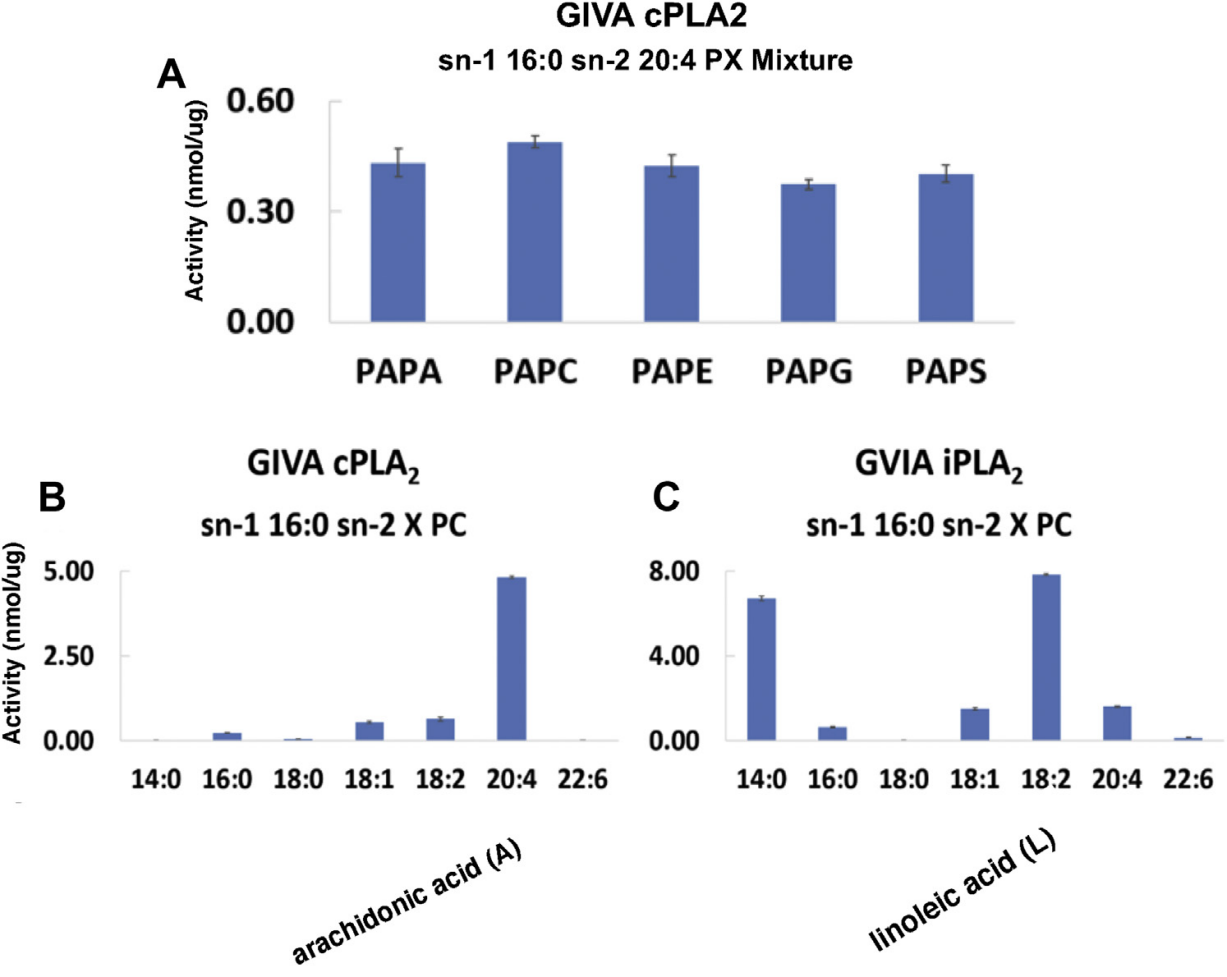
**Lipidomics connects molecular structure with cellular function.***A*, lipidomics assay of GIVA cPLA_2_ toward an equimolar mixture of five phospholipid substrates, all with palmitic acid (P; 16:0) in the *sn*-1 position and arachidonic acid (A; 20:4) in the *sn*-2 position, varying only in the polar moiety including phosphatidic acid (PA), phosphatidylcholine(PC), phosphatidylethanolamine (PE), phosphatidylglycerol (PG), and phosphatidylserine (PS) in mixed micelles with Triton X-100. *B*, acyl-chain specificity of GIVA cPLA_2_ toward *sn-2* fatty acid in PXPC, where X represents the specific fatty acid in the *sn-2* position indicated. *C*, acyl-chain specificity of GVIA iPLA_2_ toward *sn-2* fatty acid in PXPC, where X represents the specific fatty acid in the *sn-2* position indicated. cPLA_2_, cytosolic phospholipase A_2_; PAPC, *sn*-1 palmitoyl, *sn*-2 arachidonyl phosphatidylcholine. Adapted from the study by Mouchlis *et al.* (3).

We recently developed a new “substrate lipidomics” assay for PLA_2_ which allows us to look at any natural or synthetic phospholipid alone or in mixtures, rather than relying on the traditional specifically radioactively labeled phospholipids (4). Figure 8*A* shows the results of a cPLA_2_ assay of an equimolar mixture of five phospholipids, each with the same *sn-1* palmitic acid and *sn-2* AA, but varying in their polar group including zwitterionic and anionic head groups, in mixed micelles with a nonionic surfactant. The specific activity of the enzyme is the same toward all five substrates within experimental error. This observation was counterintuitive because previously the phospholipases were often identified with the kind of phospholipid that they hydrolyze based on the polar group. However, here it seems that the specificity is not with the polar group, but rather it is dictated by the specific acyl chain in the *sn-2* position that is the leaving group.

This is shown in Figure 8*B* where for cPLA_2_, the best fatty acid on the *sn-2* position is AA by far. However, when we compared the same fatty acids with iPLA_2_ in Figure 8*C*, linoleic acid was by far the best substrate and while the enzyme worked somewhat on AA, it was poorer than on linoleic acid.

**Figure 9:**
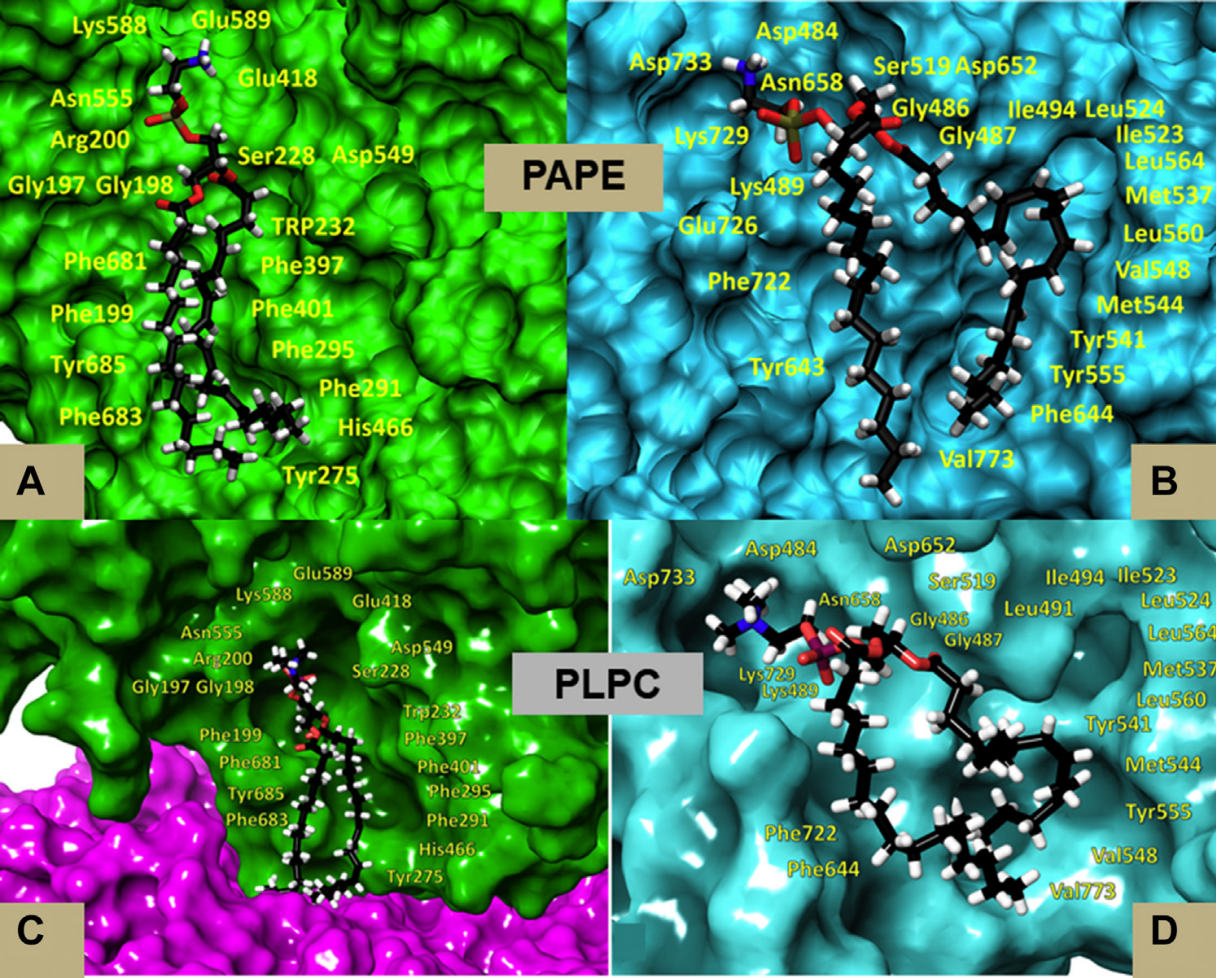
**Optimal binding conformation of enzyme and substrate after molecular dynamics simulations.***A* and *B*, the arachidonic acid in the *sn*-2 position of PAPE bound in the *sn-2* subsite of (*A*) cPLA_2_ (*green*) interacting with several aromatic amino acid side chains and (*B*) iPLA_2_ (*blue*) in a very different conformation interacting with fewer aromatic amino acids side chains after 1 μs molecular dynamics simulation. Linoleic acid in *sn*-1 palmitoyl, *sn*-2 linoleoyl phosphatidylcholine (PLPC) docked in the *sn-2* Subsite of (*C*). cPLA (*green*) at the beginning of the molecular dynamics simulation after which it dissociates into the phospholipid membrane bilayer (*purple*) and (*D*). iPLA (*blue*) interacting with several aromatic amino acid side chains after 1 μs molecular dynamics simulation. cPLA, cytosolic phospholipase A_2_; iPLA, calcium-independent phospholipase A_2_. Adapted from the study by Mouchlis *et al.* (3).

This can be explained if we look at the results of 1-μs MD simulations and the conformation of the phospholipids in the catalytic site. Shown in Figure 9*A* is cPLA_2_ with 1-palmitoyl, 2-arachidonyl phosphatidylethanolamine (PAPE) where the polar group is surrounded by charged and polar amino acids (3). That is why the active site accommodates all the different polar groups so well and apparently almost equally. But the specificity is in the arachidonyl group in the *sn-2* position, where it has its four double bonds that are all *cis*, arranged such that they interact and cause great π-π stacking with the aromatic side chains that are pictured in this catalytic site. In stark contrast as shown in Figure 9*B*, for iPLA_2_ with the same PAPE substrate, the AA is found bound with some specificity, but in a “scrunched” or not-ideal “curved” conformation. For both enzymes, the *sn-1* palmitic acid is found localized in a distinct subsite.

This can be understood and explained more clearly if the association of these two enzymes with the phospholipid substrate *sn*-1 palmitoyl, *sn*-2 linoleoyl phosphatidylcholine (PLPC) containing a *sn-2* linoleic acid is compared. Figure 9*D* shows the same subsite for iPLA_2_ with linoleic acid in the phospholipid chain, and the two *cis* double bonds in linoleic acid are aligned very nicely with aromatic tyrosine side chains and perfect π-π stacking, explaining why it binds even better than AA.

However, when the same PLPC phospholipid is docked in the active site of cPLA_2_ in the same manner that PAPE was docked and the 1-μs simulation is initiated, the phospholipid immediately diffused from the active site and moved into the membrane. See Figure 9*C*. In other words, the difference in having two less carbons and two less double bonds in linoleic acid (18:2) than AA (20:4) was enough to reduce the affinity of this fatty acid chain dramatically so that it no longer could bind over any reasonable time period within the active site.

## Secreted and lipoprotein-associated PLA_2_s

Our laboratory has used similar approaches to study the human Group V secreted sPLA_2_, which is a much smaller 13-kDa protein containing seven disulfide bonds and which utilizes a His–Asp for hydrolysis along with a required Ca^2+^. Its specificity favors linoleic acid in its *sn-2* position like iPLA_2_ (3), and most of the *sn-1* fatty acyl chain remains associated with the membrane. However, despite its differences from the two cytosolic enzymes discussed earlier in the study, it appears to follow the same general characteristics in its interaction with substrate phospholipid and membranes (3). Current work in our laboratory is aimed at more fully characterizing the unique specificity of the secreted enzyme for both the *sn-1* and *sn-2* fatty acyl groups and comparing it in more detail with that of the c- and i-PLA_2_s.

DXMS has shown that the human Group VIIA lipoprotein-associated PLA_2_ undergoes a conformational change when it associates with phospholipid vesicles and many additional changes when associated with human high-density lipoproteins (21, 22). This enzyme does not hydrolyze “normal” saturated, unsaturated, or polyunsaturated fatty acids but rather has great specificity for very-short-chain fatty acids in the *sn-2* position such as acetate (in PAF) and oxidized fatty acids such as oxovaleryl phosphatidylcholine. However, this 45-kDa extracellular enzyme utilizes a catalytic triad consisting of Ser His Asp in carry out hydrolysis. Current work in our laboratory is aimed at more fully characterizing the unique specificity of this enzyme for the *sn-2* fatty acyl groups.

## MD simulations reveals specificity of inhibitors for PLA_2_s based on the *sn*-2 subsite

Our laboratory has spent considerable effort over the years designing and studying a variety of inhibitors with specificity for each of the PLA_2_s, often in collaboration with Professor George Kokotos from Athens. Refer to the study by Dennis *et al.* (5) for an extensive review of our and other laboratories’ development of specific inhibitors through 2011, and for more recent updates, refer to the studies by Kokotou *et al.* (23) and Niolaou *et al.* (24). We recently applied our lipidomics assay for PLA_2_s to kinetically characterizing one of the optimal specific inhibitors for each of the three main PLA_2_s described in this review (4), namely pyrrophenone, which is a widely used pyrrolidine GIVA cPLA_2_ inhibitor; octylthiotrifluorophosphonate, which is a Group VIA iPLA_2_ inhibitor; and Ly315920, which is an indole developed as a Group IIA sPLA_2_ inhibitor, but which also potently inhibits Group V sPLA_2_.This study showed that the lipidomics assay works extremely well and enlarges enormously the range of phospholipid substrates available to replace the traditional radioactive-based PLA_2_ assay on PAPC that was used over the years, but for which commercial sources of substrate are no longer available.

Earlier, we extended our DXMS coupled with MD simulations to study PLA_2_ inhibitors (19, 25). With cPLA_2_, we examined the then best traditional inhibitor pyrrophenone and a designed substrate analogue oxoamide and concluded that the oxoamide binds in the substrate site with the polar moiety in the polar subsite and the alkyl chain in the *sn*-2 subsite, but the pyrrophenone bound more distal from the active site (26). However, with iPLA_2_, we discovered that for an aromatic trifluoroketone, the trifluoroketone moiety bound in the polar subsite, but the aromatic group bound in a distinct region where it was in π-π contacts with the enzyme’s aromatic sidechains, whereas an alkyl trifluoroketone bound in an adjacent region in contact with the enzyme’s aliphatic sidechains (25, 27, 28). We postulated that these two inhibitors bound in distinctive parts of the hydrophobic acyl chain sites differently if they were analogues of saturated or unsaturated chains. In our recent work on substrate specificity (3) described in Figure 9*D*, where the optimal binding of an sn-2 linoleic acid (L) in PLPC was described in close contacts with the enzyme’s aromatic side chains, we had also observed that an *sn*-2 myristic acid (M) in *sn*-1 palmitoyl, *sn*-2 myristoyl phosphatidylcholine bound with close contacts in a distinct region of the same *sn*-2 subsite surrounded entirely by aliphatic sidechains in the enzyme active site. In hindsight, the two fluroketone inhibitors (aromatic and alkyl) discriminated these two parts of the *sn*-2 subsite of iPLA_2_ (27) in the same manner as the two best substrates for iPLA_2_ did.

## Conclusion

In conclusion, we have summarized the results of DXMS, MD simulations, and lipidomics analysis reported in several recent papers that have led us to suggest that a water-soluble enzyme associates with membranes, micelles, or other lipid–water interfaces, whereby the membrane causes a conformational change in the enzyme, and aided by rapid lateral diffusion of the phospholipids in the surface of the membrane, a single phospholipid is sucked up into the catalytic site. Furthermore, each enzyme has a very well-defined and unique *sn-2* leaving fatty acid subsite. This hydrophobic *sn-2* subsite differs dramatically for each PLA_2_ and affords the unique leaving group specificity to it. This subsite results in dramatically different specificity of each PLA_2_. In contrast, the polar site, which is rather similar for each enzyme in terms of predominately charged and polar sidechain amino acids, has less specificity.

Furthermore, the *sn-1* subsite has to bind saturated fatty acid sidechains mainly consisting of palmitic acid (16:0) and stearic acid (18:0) for which the length of the fatty acid chain is the only difference, though sometimes the sn-1 position is occupied by monounsaturated and polyunsaturated fatty acids. For some of the enzymes, particularly for sPLA_2_, this is a rather shallow site and most of the fatty acid chain resides in the membrane. This concept is consistent with the overall finding that potent PLA_2_ inhibitors exhibit *Type* and *Group* specificity based on the uniqueness of the hydrophobic site at the *sn*-2 position, and this is where catalysis occurs for PLA_2_s. For all of these enzymes, it appears that as soon as catalysis occurs, the products diffuse into the membrane and the cycle repeats itself as indicated in Figure 4. In short, this manuscript has summarized the evidence that membranes allosterically activate enzymes, that lipidomics can identify substrate specificity, and that a specific *sn-2* hydrophobic subsite in each enzyme determines its unique specificity.

## Supporting information

This article contains supporting information.

## Conflict of interest

The authors declare that they have no conflicts of interest with the contents of this article.

## Supporting information

Movie S1 **MD simulation of GIVA cPLA**_**2**_**with PAPC and POPC membrane.** Molecular dynamics simulation of GIVA cPLA_2_ with PAPC docked in the active site and associated with a POPC bilayer membrane (*purple*) in accord with the results of deuterium exchange mass spectrometry. Note for visual clarity only a small number of the membrane POPC molecules is shown. Adapted from the study by Mouchlis *et al.* (19)

Movie S2 **SMD simulation of GIVA cPLA**_**2**_**extracting PAPC into the active site.** After the optimal binding mode of PAPC in the active site of GIVA cPLA_2_ was determined by molecular dynamics simulations as illustrated in Movie S1, the PAPC was pulled out of the active site along a trajectory toward the POPC bilayer membrane where it would have initially been associated in accord with the results of deuterium exchange mass spectrometry. The movie illustrates how the PAPC reenters the active site along that same trajectory when subjected to a small force in the reverse direction. Adapted from the study by Mouchlis *et al.* (19)

Movie S3 **MD simulation of GVIA iPLA**_**2**_**with PAPC and POPC membrane.** Molecular dynamics simulation of GVIA iPLA_2_ with PAPC docked in the active site and associated with a POPC bilayer membrane in accord with the results of deuterium exchange mass spectrometry. Note for clarity in observing the conformational change that iPLA_2_ exhibits during the 300-ns simulation, the membrane POPC molecules are not shown. Adapted from the study by Mouchlis *et al.* (19)
